# Changes in B Cell Populations and Merozoite Surface Protein-1-Specific Memory B Cell Responses after Prolonged Absence of Detectable *P. falciparum* Infection

**DOI:** 10.1371/journal.pone.0067230

**Published:** 2013-06-27

**Authors:** Cyrus Ayieko, Alexander C. Maue, Walter G. Z. O. Jura, Gregory S. Noland, George Ayodo, Rosemary Rochford, Chandy C. John

**Affiliations:** 1 Maseno University, Maseno, Kenya; 2 SUNY Upstate Medical University, Syracuse, New York, United States of America; 3 University of Minnesota Medical School, Minneapolis, Minnesota, United States of America; 4 Kenya Medical Research Institute, Kisian, Kenya; Université Pierre et Marie Curie, France

## Abstract

Clinical immunity to malaria declines in the absence of repeated parasite exposure. However, little is known about how B cell populations and antigen-specific memory B cells change in the absence of *P. falciparum* infection. A successful indoor residual insecticide spraying campaign in a highland area of western Kenya, led to an absence of blood-stage *P. falciparum* infection between March 2007 and April 2008. We assessed memory B cell responses in 45 adults at the beginning (April 2008) and end (April 2009) of a subsequent 12-month period during which none of the adults had evidence of asymptomatic parasitemia or clinical disease. Antibodies and memory B cells to the 42-kDa portion of the merozoite surface protein-1 (MSP-1_42_) were measured using ELISA and ELISPOT assays, respectively. B cell populations were characterized by flow cytometry. From 2008 to 2009, the prevalence of MSP-1_42_-specific memory B cells (45% vs. 55%, respectively, *P* = 0.32) or antibodies (91% vs. 82%, respectively, *P* = 0.32) did not differ significantly, although specific individuals did change from positive to negative and vice versa, particularly for memory B cells, suggesting possible low-level undetected parasitemia may have occurred in some individuals. The magnitude of MSP-1_42_-specific memory B cells and levels of antibodies to MSP-1_42_ also did not differ from 2008 to 2009 (*P*>0.10 for both). However, from 2008 to 2009 the proportions of both class-switched atypical (CD19+IgD-CD27-CD21-IgM-) and class-switched activated (CD19+IgD-CD27+CD21-IgM-) memory B cells decreased (both *P*<0.001). In contrast, class-switched resting classical memory B cells (CD19+IgD-CD27+CD21+IgM-) increased (*P*<0.001). In this area of seasonal malaria transmission, a one- year absence of detectable *P. falciparum* infection was not associated with changes in the prevalence or level of MSP-1_42_ specific memory B cells, but was associated with major changes in overall memory B cell subsets.

## Introduction

Clinical immunity to *Plasmodium falciparum* malaria is acquired and maintained by repeated exposure to the parasite [1]. Classic studies have demonstrated that passively transferred immunoglobulin G from semi-immune adults with repeated prior exposure to *P. falciparum* infection can clear or reduce parasitemia in individuals acutely infected with *P. falciparum* [2]. However, the mechanisms by which antibody secreting cells are induced and maintained for long-term disease protection are poorly understood. Repeated parasite exposure provides protection against severe disease in adults and older children in high transmission settings. This generally correlates with an age-related increase in antibody levels to blood stage *Plasmodium* vaccine-candidate antigens such as merozoite surface protein-1 (MSP-1) [3]–[5]. Studies in children have shown that antibodies to various *P. falciparum* antigens, including MSP-1, are short-lived [6]–[7], suggesting a defect in induction and maintenance of long-lived specific plasma cells to *P. falciparum*. However, studies in adults from Thailand and Madagascar done years after the most recent exposure to *P. falciparum* demonstrated the presence of long-lived memory B cell (MBC) populations and antibodies to *P. falciparum* antigens, including MSP-1 [8]–[9]. The dynamics of *P. falciparum* antigen-specific memory B cell responses over time and their alteration with changes in transmission intensity have not been studied to date.

Data on the kinetics of overall memory B cell changes during periods of prolonged lack of exposure to *P. falciparum* are also limited. Alterations in antigen stimulation from a repeated, strong immune stimulus like *P. falciparum* infection may lead to changes in the overall B cell population. Prior studies have demonstrated alterations in the proportions of B cell subsets in peripheral circulation following *Plasmodium* infections in children [10]–[12], and other studies have shown changes in B cell subsets following clinical conditions such as systemic lupus erythematosus (SLE) and infection with human immunodeficiency virus (HIV) [13]–[14]. A recent study found that populations of CD19+CD10-CD27-CD21- atypical MBCs were expanded in people exposed to endemic malaria compared to individuals that were malaria naïve [15] or exposed to lower intensity of malaria transmission [16]. These cells also expressed the inhibitory Fc receptor homolog-4 (FcRL4) marker [15]. This B cell subset was previously described in HIV-infected individuals who were reported to have poor responses to polyclonal stimulation, and these cells were termed ‘exhausted’ MBC [17]. The authors posited that exhausted MBC may play a role in poor humoral immune responses in HIV and short-lived antibody responses to malaria-infected individuals [15]–[17].

We hypothesized that absence of persistent malaria transmission would not affect circulating antigen-specific MBC in adults but might lead to loss of atypical MBCs. To test this hypothesis, we measured both MBC responses to the vaccine candidate antigen merozoite surface protein 1 (MSP-1_42_), and B cell phenotypes in adults in a region of Kenya that experiences unstable transmission. In this region, successful indoor residual insecticide spraying campaigns in 2007 and 2008 led to an almost complete absence of transmission in the area from 2007 to 2009 [18]. Immune responses were measured in 45 adults over a 12-month period from April 2008 to April 2009 during which during which no cases of clinical malaria or asymptomatic parasitemia were detected. This period followed a 14-month period from March 2007 to April 2008 during which no clinical malaria cases were recorded in the entire site [18].

## Materials and Methods

### Study Site and Study Population

This study was conducted in Kipsamoite and Kapsisiywa, two adjacent sites in the highlands areas of North Nandi District, Kenya, which is primarily comprised of the Nandi ethnic group. The sites are located at an altitude of 1,829 m and 2,132 m above sea level respectively with a total population of ∼7,800 as of 2009. Malaria transmission at the sites is low, unstable and highly seasonal [18].

This study was part of a larger prospective study assessing changes in immunity with changes in malaria incidence. A cohort of 605 randomly selected individuals was enrolled in August 2007 [18], but the first PBMC collection for the cohort was done in April 2008. A second PBMC collection was done in April 2009. Memory B cell testing was limited to adults in the study, because of the requirement for sufficient numbers of PBMC to test. Adults who had samples collected at both times and had sufficient PBMC at both times for testing of memory B cell responses (n = 45) were included in the study. Individuals in the cohort were actively followed for episodes of clinical malaria from July 2007- April 2009. Individuals who had sufficient cells for ELISPOT and flow cytometry analysis from both time points after prior testing of T cell immune responses to *P. falciparum* antigens were selected for MBC testing. Ethical clearance was obtained from Kenya Medical Research Institute, National Ethical Review Committee and the Institutional Review Board for human studies at the University of Minnesota, USA.

### Malaria Surveillance

Study participants were monitored for clinical malaria by active surveillance. During weekly visits by a trained village health worker, malaria symptoms were monitored. Any participant who reported symptoms was referred to their local dispensary for further evaluation and treatment. Malaria was treated according to Kenya Ministry of Health regulations, using artemether-lumefantrine, which has been used as first-line treatment for uncomplicated malaria in the study area since October 2006 (Kapsisiywa) and February 2007 (Kipsamoite). To test for asymptomatic infection, finger prick blood samples were obtained from participants in April, August, and October 2008 and January and April 2009. The presence of malaria parasites in the blood was assessed by microscopy as previously described [18].

### Sample Collection and Processing

Blood (10–20 ml) was drawn in heparinized vacutainers (BD, Plymouth, UK) and transported to the laboratory for processing within 6 hours of collection. Peripheral blood mononuclear cells (PBMC) were separated by Ficoll-hypaque density gradient centrifugation and cryopreserved in liquid nitrogen at a concentration of 10^7^ cells/ml in heat-inactivated 90% fetal bovine serum (Sigma, St Louis, MO) and 10% DMSO (Sigma, St Louis, MO). Plasma samples were stored at −20°C for antibody analysis.

### Antibody Testing

Recombinant MSP-1_42_ (3D7 strain) produced in *E. coli*, and provided courtesy of David Narum of the National Institutes of Health, was used for all MSP-1_42_ testing. Immulon-4 plates (Dynex Technologies, Chantilly, VA) were coated with 0.1 µg/ml of recombinant MSP-1_42_ in 1xPBS or 1 µg/ml tetanus toxoid (TT, EMD Bioscience, San Diego, CA) respectively. After overnight incubation at 4°C, the plates were washed twice with PBS before incubating for 1 hour at 37°C with blocking buffer: PBS plus 3% bovine serum albumin (BSA, Sigma, St Louis, MO)) and 0.05% Tween-20 (Sigma, St Louis, MO). Plasma samples diluted at 1∶100 in blocking buffer were loaded onto the plates in duplicates and incubated for 1 hour at 37°C. After washing four times with wash buffer (PBS plus 0.05% Tween-20), alkaline phosphatase–conjugated goat anti-human IgG (Jackson ImmunoResearch, West Grove, PA) diluted 1∶1,000 in blocking buffer was added to the plates and incubated for 1 hour at 37°C. The plates were then washed four times with wash buffer and developed by adding alkaline phosphatase substrate (Sigma, St Louis, MO) before reading the optical density (OD) at 405 nm (Molecular Devices, Sunnyvale, CA). Plasma samples from 8 malaria-naïve North American adults were included in each of the MSP-1-coated plates as negative controls. Antibody levels for MSP-1_42_ responses were expressed in arbitrary units (AU), which were calculated by dividing the test sample ODs by the mean OD +3 standard deviations (SDs) from North Americans control sera. Individuals with antibody levels of >1 AU were considered positive responders. The cut-off for TT-specific antibody responses was set at >0.2, based on the frequency distribution of untransformed ODs.

### B cell ELISPOT

Antigen-specific memory B cells were quantified using the ELISPOT technique developed by Crotty *et al* [19] and modified by Weiss *et al* [20], [21]. Briefly, frozen PBMCs were quick thawed in a 37°C water bath and washed with PBS containing 10% fetal bovine serum (FBS, Sigma, St Louis, MO) and 1% penicillin-streptomycin (Sigma, St Louis, MO). Viable cells were counted and suspended at 10^6^ cells/ml in complete media (RPMI 1640 (Biowhittaker, Walkersville, MD) with 10% heat inactivated-FBS (Sigma, St Louis, MO), and 50 µM β-mercaptoethanol (Sigma, St Louis, MO) plus a cocktail of the following polyclonal activators: 2.5 µg/ml of CPG ODN-2006 (Operon Technologies, Huntsville, AL), 1∶10,000 dilution of *Staphylococcus aureus* Cowan protein A (Sigma, St Louis, MO), 50 ng/ml of pokeweed mitogen (Sigma, St Louis, MO) and IL-10 (R&D Systems, Minneapolis, MN ) at 25 ng/ml. The cells were cultured at 1 × 10^6^ cells per well in 24-well tissue culture plates (Corning Inc., New York) at 37°C, 5% CO_2_ for 5 days. On the 6^th^ day, cells were harvested by repeated pipetting, and washed in warm complete media in preparation for plating onto antigen/antibody-coated ELISPOT plates.

ELISPOT plates (Millipore Multiscreen-HA) were coated with either polyvalent goat anti-human IgG (Invitrogen, Carlsbad, CA) at 10 µg/ml in PBS or recombinant MSP-1_42_ (3D7 allele) at 2.5 µg/ml in PBS. Additional wells were coated only with TT at 2.5 µg/ml or 1% BSA in RPMI-1640 as positive and negative controls, respectively. After an overnight incubation, plates were blocked with 1% BSA in RPMI-1640 for 2 h at 37°C. Polyclonally stimulated cells were then plated in duplicate onto anti-human IgG-coated plates at 1 × 10^4^ cells per well. Cells were plated in duplicate into TT-coated and MSP-1_42_ -coated plates at 5×10^5^ cells per well, and then serially diluted 1∶2. BSA-coated wells were seeded with 5×10^5^ cells per well. Plates were kept at 37°C in a 5% CO2 incubator for 6 h. After four washes with PBS, and four washes with wash buffer, goat anti-human IgG Fc-alkaline phosphatase diluted (Jackson ImmunoResearch, West Grove, PA) diluted 1∶1,000 in wash buffer plus 1% FBS was added to wells and incubated overnight at 4°C. Plates were washed four times with wash buffer, three times with PBS, and three times with distilled water, before developing with the substrate BCIP/NBT (Sigma, St Louis, MO). The plates were finally washed in deionized water to stop the reaction. Spots were counted with the ImmunoSpot series 3.2 analyzer (Cellular Technology, Cleveland, OH). Antigen specific MBCs were expressed as the number of Ag-specific MBCs per million PBMCs divided by number of IgG-secreting cells per million PBMCs × 100 [19]. A well was scored as positive if it had at least twice the number of spots as the negative control wells (BSA-coated wells) [9].

### Immunophenotyping of Peripheral Blood Lymphocytes

Cryopreserved PBMCs were quickly thawed in a water bath at 37°C and washed in cold flow buffer (PBS plus 1% BSA (Sigma, St Louis, MO) and 0.1% sodium azide (Sigma, St Louis, MO). The PBMCs were stained with Trypan blue and enumerated using a hemocytometer. For B cell phenotyping, fluorochrome-conjugated mouse antihuman monoclonal antibodies were used to stain 10^6^ cells/100 µl flow buffer. A cocktail consisting of the following mouse antihuman antibodies was used: PerCP-Cy5.5-IgM, PE-FcRL4 (Biolegend, San Diego, CA), APC-eFluorTM780-CD27(eBioscience, San Diego, CA), FITC–IgD, APC-CD21, AlexaFluor700-CD19 (BD Bioscience, Franklin Lakes, NJ). After staining for 30 minutes, cells were washed then incubated for 5 minutes with 4′, 6-diamidino-2-phenylindole (DAPI) (Invitrogen, Carlsbad, CA) to allow for gating on viable cells (DAPI-negative). After two washes with flow buffer the cells were fixed for 15 minutes with 1% paraformaldehyde (Sigma, St Louis, MO), before finally resuspending in 300 µl flow buffer. Analysis was done on a LSRII flow cytometer (Becton Dickinson Immunocytometry Systems, San Jose, USA). Data was processed using FLOWJO software (Tree Star Inc., San Carlos, Ca, USA).

### Statistical Analysis

The prevalence of antigen-specific memory B cells and antibodies within the same individuals were compared by McNemar’s test, and magnitude of memory B cells and antibody levels within the same individuals were compared by the Wilcoxon matched pairs test. Correlations between MSP-1_42_ specific antibody levels (ELISA) and the magnitude of MSP-1_42_ specific memory B cells (ELISPOT) were calculated by Spearman’s rank test. Mean percentages of B cell frequencies between time points in the same individuals were compared by paired t-tests. Stata version 10.0 (Stata Corporation, TX) was used to analyze the data.

## Results

### Characteristics of the Study Population

The study was conducted in a highland population in western Kenya with a history of unstable malaria transmission, but which had had no malaria transmission for at least 14-months [18]. To analyze B cell phenotype and malaria specific immunity in the absence of persistent malaria infection, samples from forty-five adults (mean age = 38.8 years; range 17.8–66.3 years; females = 58%) were collected at two time points over a 12 months period (April 2008 and April 2009). None of the 45 participants had an episode of clinical malaria during the one-year investigation period or in the preceding eight months, and all tested negative for *P. falciparum* by microscopy in repeated surveys for asymptomatic parasitemia before (July 2007 and November 2007) and during (April 2008, August 2008, October 2008, January 2009 and April, 2009) the twelve-month study period. B cell phenotyping was done on all participants but only forty had sufficient cells to measure MSP-1_42_ -specific MBCs by ELISPOT.

### No Change in Antibody Prevalence or Levels to MSP-1_42_ or Tetanus Toxoid Over Time

**Figure 1:**
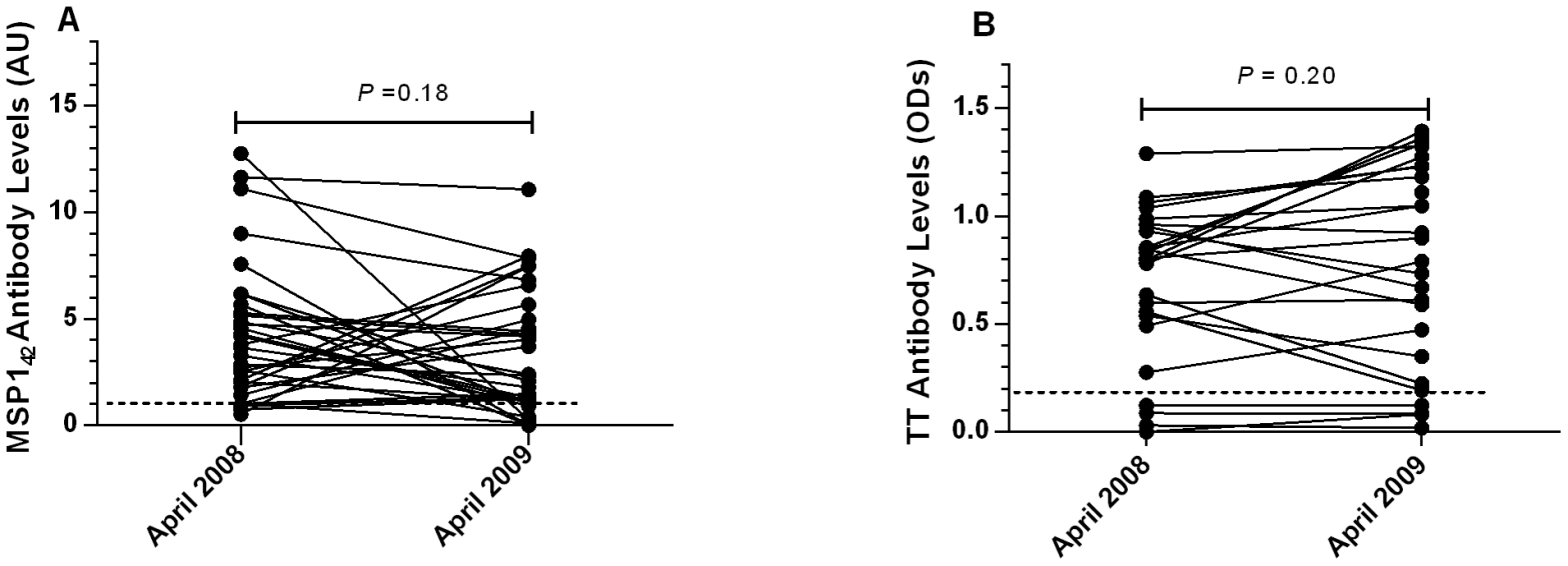
Serum antibody levels of merozoite surface protein-1_42_ (MSP1_42_)-specific (Figure 1A) and tetanus toxoid (TT)-specific (Figure 1B) antibodies. Individuals with antibody levels >1 AU and >0.2 OD were considered responsive to MSP-1_42_ and TT, respectively.

Episodes of *P. falciparum* malaria have been previously recorded in our study area [22]. This region of Kenya experiences intermittent transmission of *P. falciparum*; within the last several years, increased malaria control has reduced the transmission of malaria in this area. To confirm previous exposure to malaria, we tested our study population for antibodies to MSP-1_42_ by ELISA. In 2008, 91% of individuals tested had antibodies to MSP-1_42._ Although the sero-prevalence declined to 82% in 2009, this difference was not significant (*P = *0.32, Table 1 ). Six individuals who were positive for MSP-1_42_ antibodies in 2008 became negative in 2009 while three individuals who were seronegative in 2008 became seropositive (Table 1). Levels of MSP-1_42_ -specific antibodies also did not differ significantly between 2008 (median (arbitrary units [AU]), 3.64, range, 0.52–12.76) and 2009 (median (AU), 2.37, range, 0.0–11.07, *P* = 0.18, Figure 1A). The percentage of individuals with antibodies to the control antigen TT was likewise similar between 2008 (84%) and 2009 (84%, *P* = 1.00, Table 2), as were TT antibody levels in 2008 (median (OD), 0.81, range, 0.0–1.29) and 2009 (median (ODs), 0.79, range, 0.02–1.39; *P* = 0.20, Figure 1B).

**Table 1:** Prevalence of Immunoglobulin G (IgG) antibodies to the merozoite-surface protein-1 (42) (MSP-1_42_) in highland Kenyan adults in 2008 and 2009.

		MSP-1_42_ IgG Ab,2008		
		Positive	Negative	Total
MSP-1_42_ IgG Ab,2009	Positive	24 (73%)	3 (9%)	27 (82%)
	Negative	6 (18%)	0 (0%)	6 (18%)
	Total	30 (91%)	3 (9%)	33 (100%)

McNemar’s test, *P* = 0.32.

**Table 2:** Prevalence of toxoid (TT)-specific immunoglobulin G (IgG) antibodies in highland Kenyan adults in 2008 and 2009.

		TT IgG Ab, 2008		
		Positive	Negative	Total
TT IgG Ab, 2009	Positive	21 (84%)	0(0%)	16 (84%)
	Negative	0(0%)	4 (16%)	4 (16%)
	Total	21 (84%)	4 (16%)	25 (100%)

McNemar’s test, *P* = 1.0.

### No Change in Magnitude of Memory B cells to MSP-1_42_ or Tetanus Toxoid Over Time

To determine if there were changes in the magnitude of *Plasmodium*-specific memory B cells between 2008 and 2009, frozen PBMC were tested using B cell ELISPOT. Eighteen of the forty individuals tested (45%) had MBCs to MSP-1_42_ in 2008 and twenty-two (55%) had MBC to MSP-1_42_ antigen in 2009 (*P* = 0.32, Table 3). Six individuals who were positive for MBC to MSP-1_42_ in 2008 became negative in 2009, while ten non-responders in 2008 gained responses in 2009 (Table 3). Although nineteen individuals were tested for TT, one individual had an extremely high magnitude of MBC (35.91%). These results suggested a possible error in the assay procedure and so were excluded. Of the remaining eighteen individuals, thirteen (72%) tested positive for circulating TT-specific MBC in 2008, while fourteen (78%) were positive in 2009 (*P* = 0.71, Table 4).

**Table 3:** Prevalence of memory B cells (MBC) to merozoite surface protein-1 (42) (MSP-1_42_) in highland Kenyan adults in 2008 and 2009.

		MSP-1_42_ MBC, 2008		
		Positive	Negative	Total
MSP-1_42_ MBC,2009	Positive	12 (30%)	10 (25%)	22 (55%)
	Negative	6 (15%)	12 (30%)	18 (45%)
	Total	18 (45%)	22 (55%)	40 (100%)

McNemar’s test, *P* = 0.32.

**Table 4:** Prevalence of tetanus toxoid (TT)-specific memory B cells (MBC) in highland Kenyan adults in 2008 and 2009.

		TT MBC, 2008		
		Positive	Negative	Total
TT MBC, 2009	Positive	10 (56%)	4 (22%)	14 (78%)
	Negative	3 (17%)	1 (6%)	4 (22%)
	Total	13 (72%)	5 (28%)	18 (100%)

McNemar’s test, *P* = 0.71.

**Figure 2:**
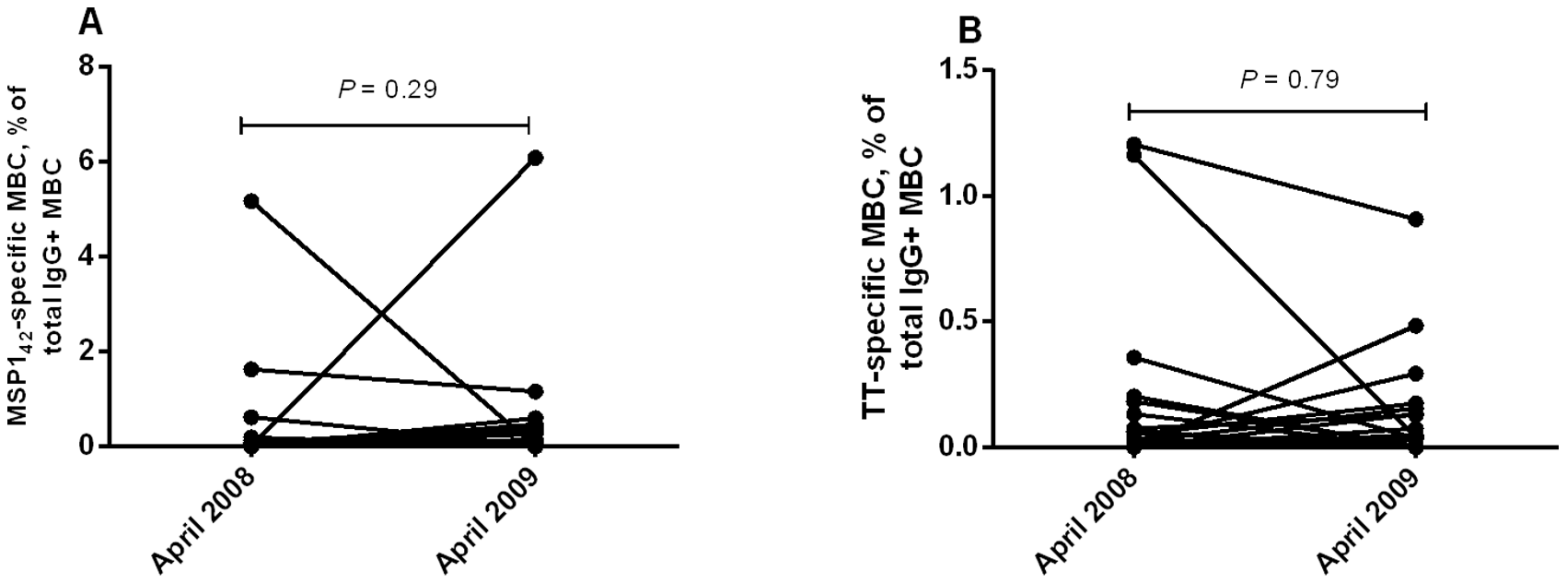
The magnitude of memory B cell (MBC) responses to the merozoite surface protein-1_42_ (MSP1_42_) and tetanus toxoid (TT) in 2008 and 2009. Magnitude of antigen specific MBCs was expressed as a percentage, calculated by diving the number of Ag-specific MBCs per million PBMCs by number of IgG-secreting cells per million PBMCs multiplied by 100. The magnitude of the MSP-1_42-_specific MBC (A) and TT-specific MBCs (B) increased significantly between 2008 and 2009.

We next determined if the levels of MSP-1_42_ specific MBCs changed over time. The magnitude of MSP-1_42_ -specific MBC as a percentage of total MBCs did not change between 2008 (median = 0%; range = 0–5.17%) and 2009 (median = 0.01%; range = 0–6.09%; *P* = 0.29) (Figure 2A). Similarly the magnitude of TT-specific MBC did not vary between 2008 (median = 0.07%; range = 0–1.39%) and 2009 (median = 0.11%; range = 0.0–4.01%; *P* = 0.79) (Figure 2B).

**Figure 3:**
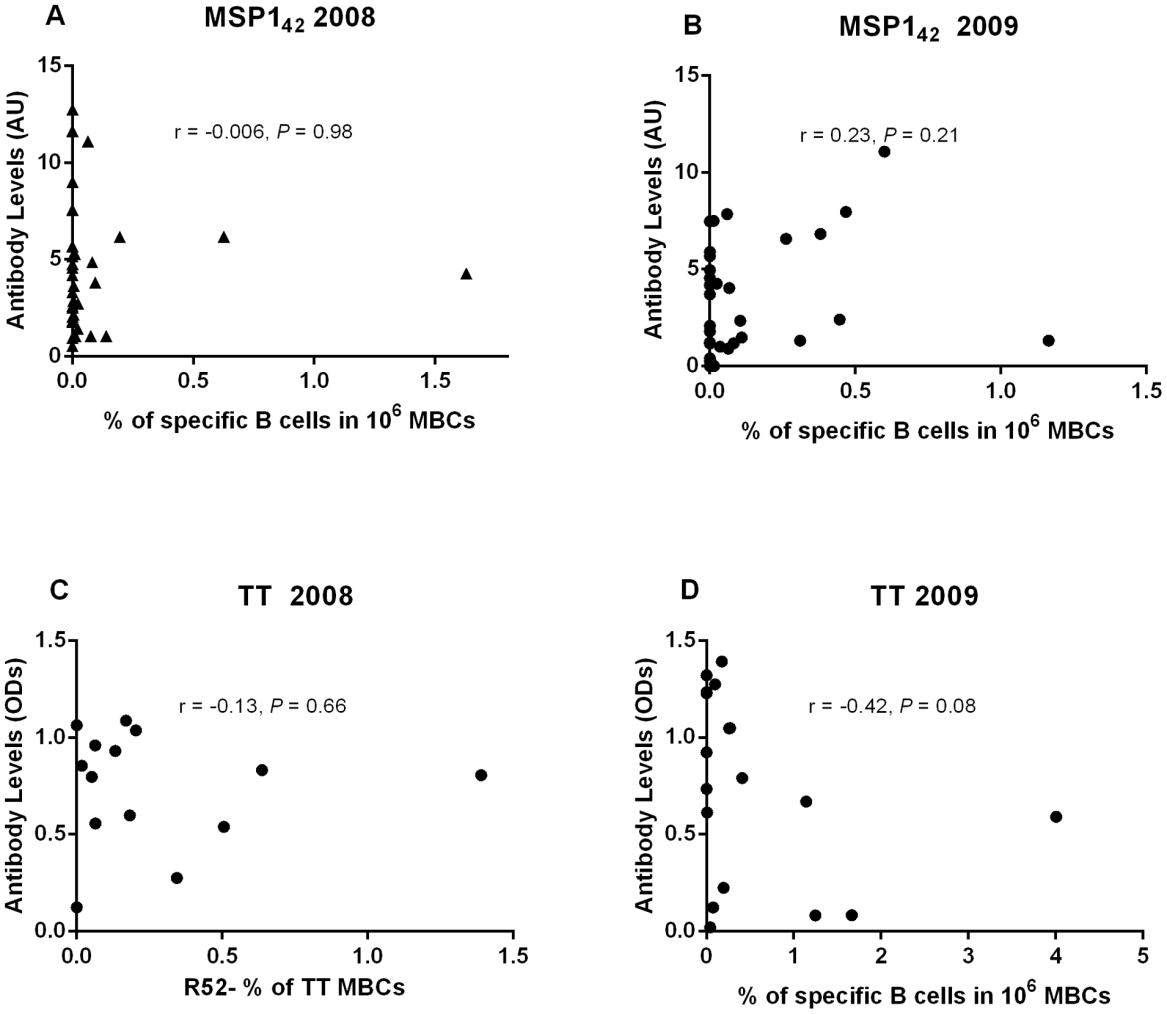
Comparison between estimated memory B cell frequencies and antibody responses to merozoite surface protein-1_42_ (MSP-1_42_) and tetanus toxoid (TT). There was no association between specific MBCs and antibodies for both MSP-1_42_ and TT in 2008 and 2009.

The percentage of individuals who had antibodies to MSP-1_42_ was higher than the percentage of individuals with detectable circulating MBCs to MSP-1_42_ in both 2008 (*P* = 0.0005) and 2009 (*P* = 0.03) (Tables 5 and 6), whereas the percentage of responders to TT as estimated by the two assays was similar at both time points (2008, *P* = 0.32; 2009, *P* = 0.71) (Tables 7 and 8). There was no correlation between antibody levels and magnitude of antigen-specific MBC for either MSP-1_42_ or TT (Figure 3).

**Table 5:** Number and percentages of responders to merozoite-surface protein-1 (42) (MSP-1_42,_ as assessed by antigen-specific memory B cells and immunoglobulin G (IgG) antibodies (Ab), in 2008.

		IgG Ab		
		Positive	Negative	Total
MBC	Positive	15 (46%)	1 (3%)	16 (49%)
	Negative	15 (46%)	2 (6%)	17 (51%)
	Total	30 (91%)	3 (9%)	33 (100%)

McNemar’s test, *P = *0.0005.

**Table 6:** Number and percentages of responders to merozoite-surface protein-1 (42) (MSP-1_42_ as assessed by antigen-specific memory B cells and immunoglobulin G (IgG) antibodies (Ab), in April 2009.

		IgG Ab		
		Positive	Negative	Total
MBC	Pos	16 (49%)	3 (9%)	19 (58%)
	Negative	11 (33%)	3 (9%)	14(42%)
	Total	27 (82%)	6 (18%)	33 (100%)

McNemar’s test, *P = *0.03.

**Table 7:** Number and percentages of responders to tetanus toxoid (TT) as assessed by antigen-specific memory B cells and immunoglobulin G (IgG) antibodies (Ab) in April 2008.

		IgG Ab		
		Positive	Negative	Total
MBC	Positive	12 (86%)	0 (0%)	12 (86%)
	Negative	1 (7%)	1 (7%)	2 (14%)
	Total	13 (93%)	1 (7%)	14 (100%)

McNemar’s test, *P = *0.32.

**Table 8:** Number and percentages of responders to tetanus toxoid (TT) as assessed by specific memory B cells and immunoglobulin G (IgG) antibodies (Ab) in April 2009.

		IgG Ab		
		Positive	Negative	Total
MBC	Positive	11 (65%)	3 (18%)	14 (82%)
	Negative	3 (18%)	0 (0%)	3 (18%)
	Total	14 (82%)	3 (18%)	17 (100%)

McNemar’s test, *P = *0.71.

### Changes in B cell Population and Subsets Over Time

**Figure 4:**
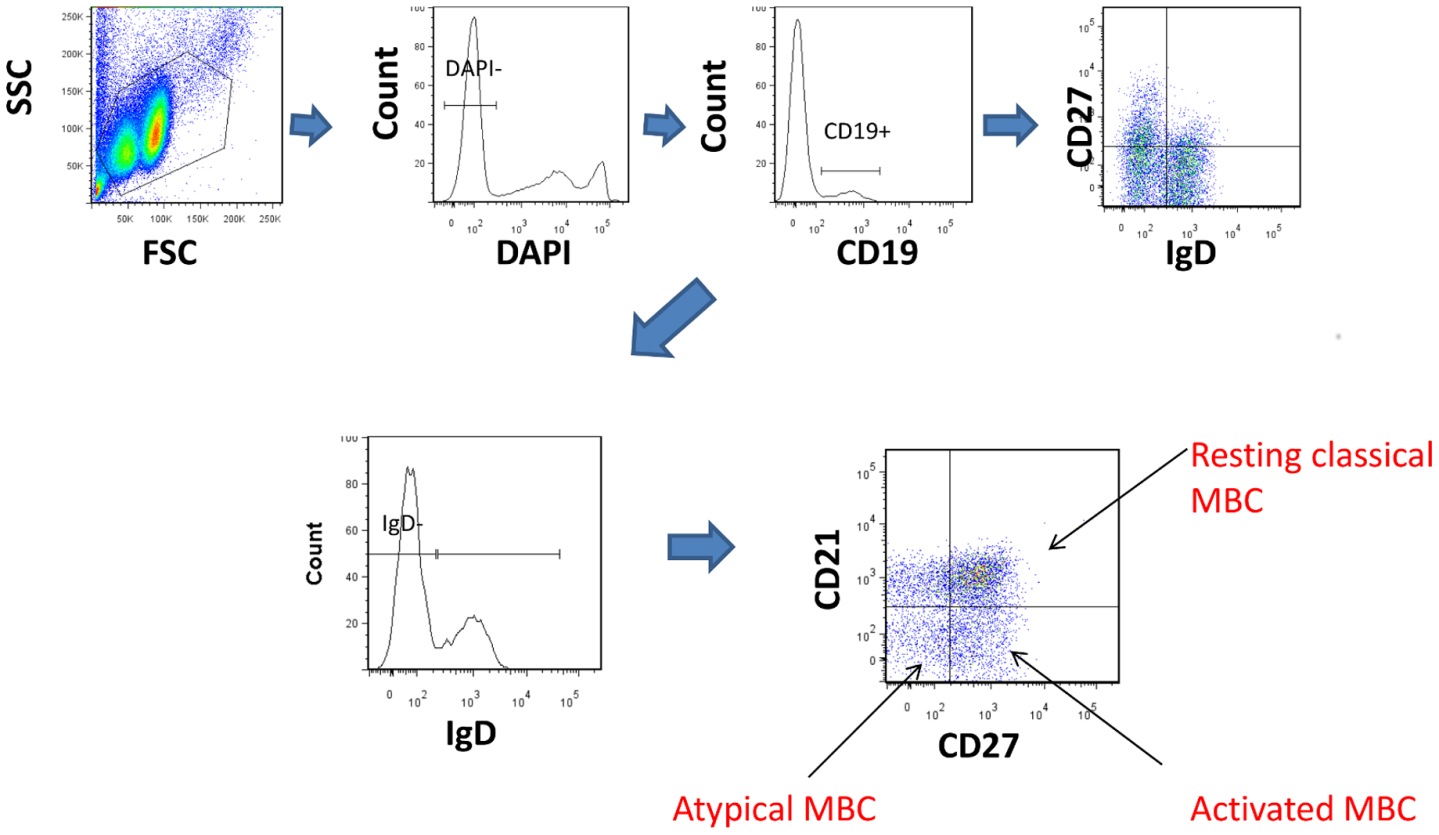
The gating strategy for identifying the B cell subpopulations. We first gated on the live (DAPI^−^) lymphocytes and then collected 10,000 events of CD19+. These were analyzed for expression of IgD, CD27, CD21, IgM and FcRL4. CD19+ lymphocytes were classified as naïve (IgD+CD27-) B cells, non class-switched (IgD+CD27+) MBCs, classical (IgD-CD27+) MBCs and double negative (IgD-CD27-) MBCs. MBC subtypes were further defined as follows: 1. Resting classical MBCs: CD19+IgD-CD27+CD21+. 2. Activated MBCs: CD19+IgD-CD27+CD21-. 3. Atypical MBCs: CD19+IgD-CD27-CD21- MBCs.

**Figure 5:**
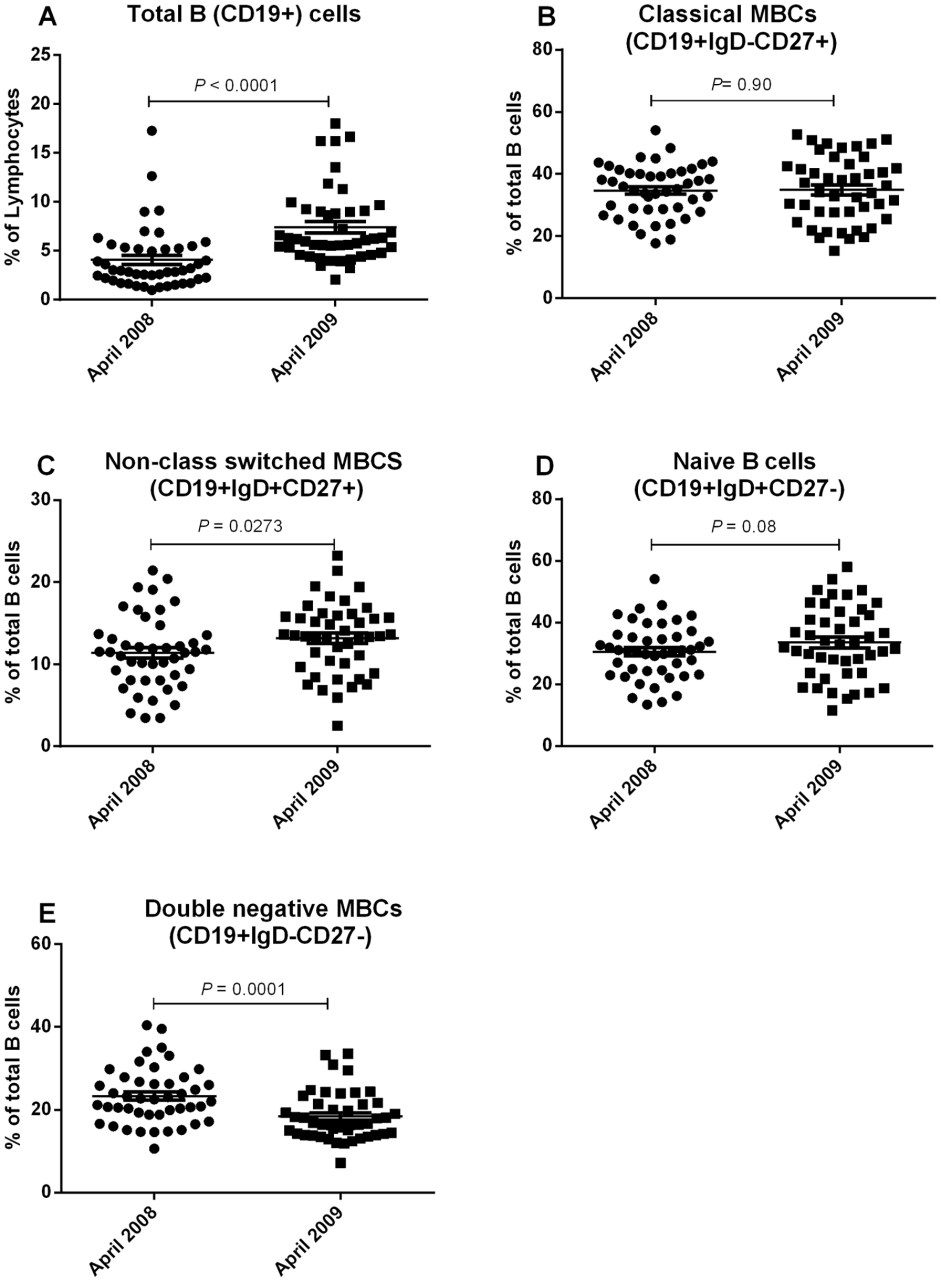
Proportion of B cells and B cell subsets in 2008 and 2009. The mean percentages of total (CD19+) B cells (A), classical (CD19+IgD-CD27+) MBCs (B), non-class switched (CD19+IgD+CD27+) MBCs (C), naïve (CD19+IgD+CD27-) B cells (D) and double-negative (CD19+IgD-CD27-) MBCs (E) over the study period.

**Figure 6:**
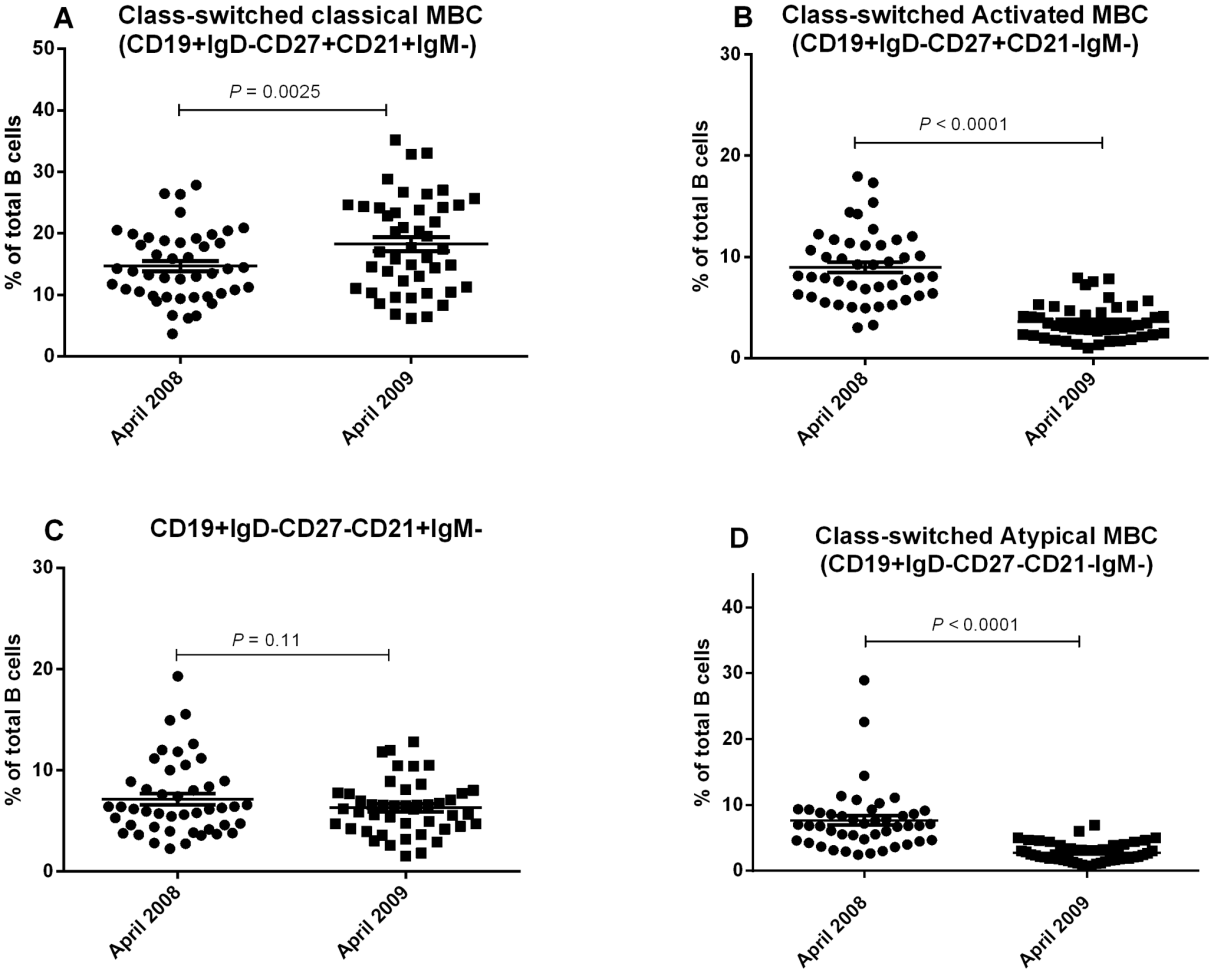
The distribution of class-switched memory B cell subsets in adults from highland Kenya. Mean percentages of: (A) class-switched resting classical (CD19+IgD-CD27+CD21+IgM-) MBCs, (B) class-switched activated classical (CD19+IgD-CD27+CD21-IgM-) MBCs, (C) class-switched CD19+IgD-CD27-CD21+ and (D) class-switched atypical (CD19+IgD-CD27-CD21-IgM-) MBCs.

Previous studies have reported elevation of atypical MBCs and suppression of non class-switched MBCs in high malaria transmission areas relative to low transmission area [11], [15], [16]. To determine if there were changes over time in B cell subsets in individuals who had been exposed to *P. falciparum* but had been free of infection for at least 12 months, we performed immunophenotypic analysis of PBMC isolated from study participants in 2008 and 2009. A representative flow analysis is shown in Figure 4 and the summary of the data is shown in Figure 5 and Figure 6.

There was an increase in the percentage of PBMC that were B cells (CD19+) between 2008 (mean, 4.06%, standard deviation (SD), 3.15%) and 2009 (mean, 7.39%; SD, 3.81%; *P*<0.0001, Figure 5A). However, the percentage of B cells that were classical MBCs (CD19+IgD-CD27+) remained constant (mean, 34.7%; SD, 8.21% in 2008 and mean, 34.88%; SD, 10.5 in 2009; *P* = 0.9, Figure 5B), while non-class switched (CD19+IgD+CD27+) MBCs increased between 2008 (mean, 11.40%; SD, 4.46) and 2009 (mean, 13.18%; SD, 4.32%; *P* = 0.0273, Figure 5C). Conversely, naïve (CD19+IgD+CD27-) B cells did not vary significantly between 2008 (mean, 30.58%; SD, 8.95) and 2009 (mean, 33.54%; SD, 11.64%; *P* = 0.08, Figure 5D). Double-negative (CD19+IgD-CD27-) MBCs decreased from 2008 to 2009 (mean, 23.33%; SD, 6.77% in 2008 and mean, 18.4%; SD, 5.82 in 2009; *P* = 0.0001, Figure 5E).

To further evaluate MBC subsets, we characterized them based on expression of IgD, CD27, CD21 and IgM. We first assessed subsets of class-switched MBC (CD19+IgD-IgM-). The percentage of class-switched resting classical MBCs (CD19+IgD-CD27+CD21+IgM-) as a percentage of total B cells increased from 2008 to 2009 (mean (SD), 2008, = 14.71 (5.68) %) vs, 2009, 18.27 (7.67) %; *P = *0.003, Figure 6A). In contrast, class-switched activated MBCs (CD19+IgD-CD27+CD21-IgM-) decreased (mean (SD), 2008 = 8.97 (3.5) %) vs. 2009, 3.59 (1.78) %; *P*<0.0001, Figure 6B). The percentage of CD19+IgD-CD27-CD21+IgM- MBCs remained constant between the two time points (mean (SD), 2008 = 7.14 (3.73) %) vs 2009, 6.31 (2.67) %; *P* = 0.11, Figure 6C). Class-switched atypical (CD19+IgD-CD27-CD21-IgM-) MBCs decreased from 2008 to 2009 (mean (SD), 2008 = 7.66 (4.80) %) vs 2009, 2.76 (1.47) %; *P*<0.0001, Figure 6D).

In addition, we examined the proportions of class-switched resting classical MBC, activated classical MBC and atypical MBC subsets between individuals who had MSP1_42_-specific MBC and those who did not. No differences between the two groups were observed in 2008 (Figure S1) and 2009 (data not shown).

**Figure 7:**
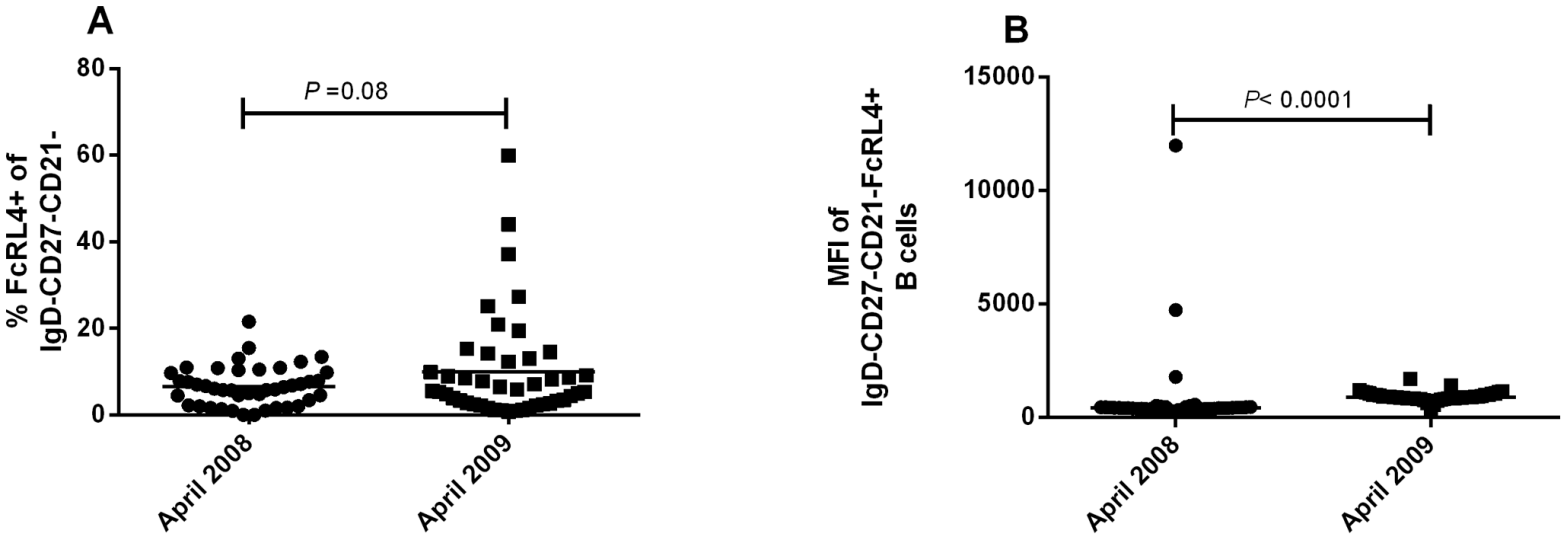
The distribution of FcRL4+ memory B cell subsets. The percentage of FcRL4+ CD19+IgD-CD27-CD21 MBCs had a non-significant increase (A) but the mean fluorescence intensity (MFI) of FcRL4+ cells increased significantly (B). Lines in 7A reflect mean values and comparison is with t-test. Lines in 7B reflect median values and comparison is with Wilcox on signed rank test because MFI values were not normally distributed.

Fc-receptor-like-4 is an immunoregulatory receptor that is up-regulated in atypical memory B cells and has been associated with B cell exhaustion [16], [17], [23]. We investigated the effect of reduced malaria transmission on the expression of this receptor on atypical MBC. The percentage of atypical MBC that expressed the inhibitory receptor FcRL4+ increased modestly but not significantly between 2008 and 2009 (Figure 7A, *P* = 0.08). Among cells that were FcRL4+, the mean fluorescence intensity (MFI) increased significantly from 2008 (median, 420.17; range 285.96–11992.42) to 2009 (median, 911.63; range 393.93–1704.64; *P*<0.0001, Figure 7B).

**Figure 8:**
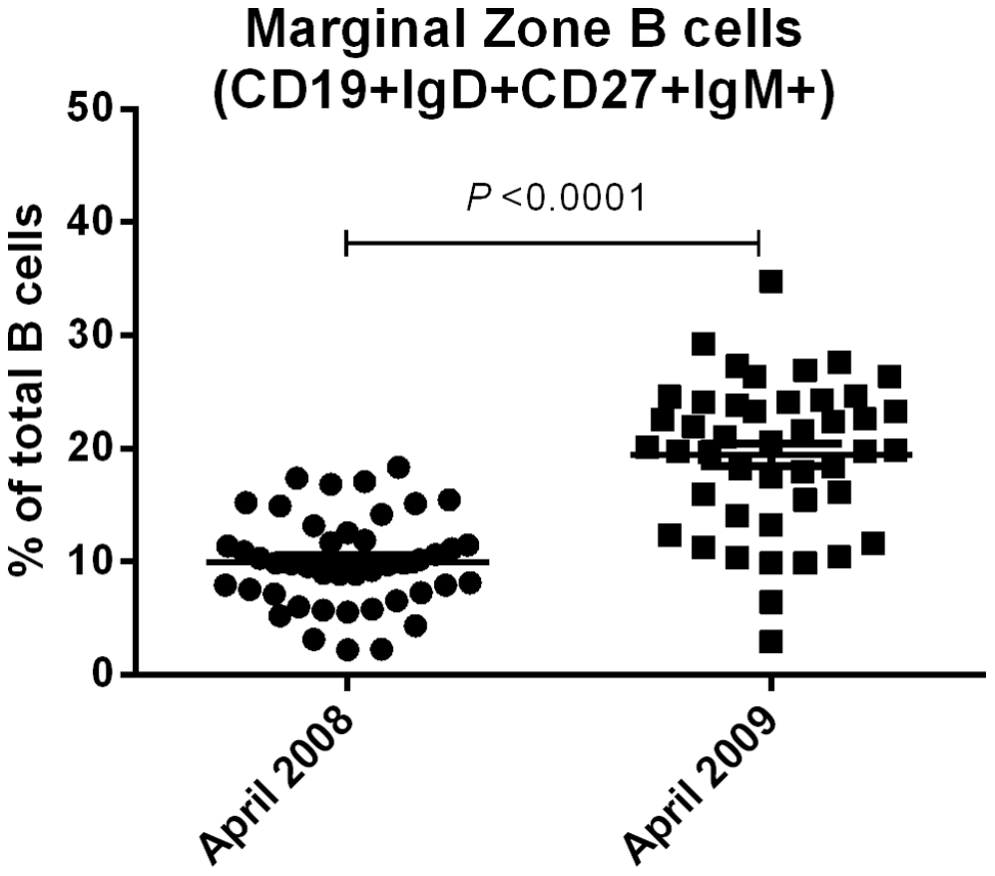
The proportions of marginal zone (CD19+IgD+CD27+IgM+) B cells increased between 2008 and 2009.

Since exposure to holoendemic malaria has been shown to suppress non-class switched (CD19+IgD+CD27+) MBC populations [11], we evaluated the effect of reduced malaria transmission on the marginal zone B cells (CD19+IgD+CD27+IgM+) [24], a subset of this B cell population. The percentage of CD19+IgD+CD27+IgM+ MBC increased from 2008 (mean, 9.95%; SD, 4.05%) and 2009 (mean, 19.45%; SD, 6.58%; *P*<0.0001, Figure 8).

## Discussion

In this study, we document that antibodies and MBCs to MSP-1_42_ are stable over a period of at least 12 months in the absence of detectable *P. falciparum* infection in previously malaria-exposed adults in a highland area of Kenya. In addition, we demonstrate that absence of detectable *P. falciparum* infection in this population is associated with major changes in B cell homeostasis, notably a decrease in atypical MBC and activated MBC subsets. These results suggest that the absence of persistent malaria transmission in a previously exposed population may lead to changes in B cell phenotypes in adult populations. This study assessed the stability of humoral immune memory and B cell phenotypes, an area little studied to date. Our future studies will assess the mechanisms underlying the changes.

Past studies have provided conflicting data on the persistence of specific-antibodies to blood-stage *P. falciparum* antigens in individuals living in malaria endemic areas. The differences may be in part due to differences in the age groups studied, the methods and epidemiological settings. Studies of children in high transmission areas have found antibodies to MSP-1 to be short-lived after resolution of infection [6]. However, in studies looking at a full spectrum of ages, antibodies to MSP-1 in older children and adults appear to persist [25] possibly 40 years or more. The present study findings are consistent with those in other adult populations, including a study done across transmission gradients [25] and a study from a low malaria transmission area in Thailand [9] that showed that antibodies to MSP-1_19_ in adults persisted long after transmission ended in that area.

There have also been conflicting data on the durability of the pool of *P. falciparum*-specific MBC after interruptions in transmission. The B cell memory findings in the present study are consistent with the findings of Wipasa *et al*, [9] who reported the persistence of specific MBCs to PfSE, PfAMA-1 or PfMSP-1_19_ after more than 5 years absence of malaria infection. In the present study, antibodies to MSP-1_42_ were more frequent than MSP-1_42_-specific MBCs at both time points. These findings differ from those of a study in coastal Kenya, in which MBCs and antibodies to MSP-1_19_ were seen in similar frequencies [26], but in the same study, antibodies to apical membrane antigen −1 (AMA-1) and a component of *Plasmodium falciparum* erythrocyte membrane protein 1 (PfEMP1) PfEMP-1 were more frequent than MBCs to these antigens. Differences in study findings may partly relate to our use of a different antigen form (MSP-1_42_ as opposed to MSP-1_19_) and a more sensitive B cell detection assay [21]. Hui *et al* [27] showed that MSP-1_42_ induced antibodies recognize many allelic forms of MSP-1_19_ as compared to antibodies to MSP-1_19_, perhaps explaining why the prevalence of antibodies were much higher in this study than found in the coastal Kenya population. In addition, Dorfman *et al* [26] enrolled 15 adults and 57 children, a population that would have different immune responses from the adults in the present study. Consistent with our findings, Ndungu *et al* recently showed that specific MBC to malaria antigens are maintained in the absence of persistent infection in previously exposed Kenyan children [28]. However, they also found that MSP-1_42_-specific MBC were a more efficient measure of past infection compared to MSP-1_42_-specific antibodies. In our study, we found that detection of past infection was more robust with antibodies. These contrasting observations likely reflect differences between immune responses in children and adults.

Memory B cells and plasma cells are central in maintaining long term antibody production, but it is not clear what is required to sustain the antibody responses to malaria antigens over time. One hypothesis is that periodic antigenic exposure maintains production and is provided by asymptomatic infection [29] or by parasite antigens sequestered on antigen presenting cell (APC) surfaces [30]. On repeated surveys, none of the individuals in this study had asymptomatic parasitemia by microscopy or PCR [18], so a high frequency of undetected parasitemia in this study group is unlikely, although the occurrence of MSP-1_42_-specific MBC in individuals in 2009 who were previously negative in 2008 suggests that some individuals may have had undetected asymptomatic parasitemia during the course of the year. Asymptomatic parasitemia could also not explain the persistence of antibody responses in the Thailand and Madagascar study sites [8]–[9] where malaria was absent in the population for several years. It is more likely that the antibody levels are maintained by long-lived plasma cells. In fact, studies in mice have reported that stable populations of long-lived B220+ plasma cells are established after a secondary *P. chabaudi* infection [31]. This could partly explain the high prevalence of antibodies to MSP-1 in this study relative to the finding in the coastal Kenya population with high malaria endemicity [26]. Similarly, a study in the Gambia during a period of low malaria transmission recorded high prevalence of antibodies to AMA-1, MSP-2 and MSP-3 [32]. Persistent malaria infection evidently intereferes with establishmment of long-lived B cell memory, probably by deleting antigen specific B cells [33] or disrupting of germinal cell architecture hence disrupting transformation of centrocytes [34]. Chronic infections may also influence the fate and lifespan of plasma cells such that a majority of plasma cells generated during inflammation express the chemokine receptor CXCR3 thus migrate into chronically inflamed tissue, forming the short-lived component of antibody response [35]. In contrast, plasma cells generated in the absence of inflammation express the CXCR4 receptor and so migrate to the bone-marrow where they persist as long-term antibody producing cells [36] without the support of antigens or MBCs [37]–[38]. Low exposure to malaria could therefore be beneficial to the establishment of long-term memory to malaria as more plasma cells develop to long-lived plasma cells.

The increase in the proportion of CD19+ B cells, and the total number of B cells, after interruption of malaria transmission was consistent with studies showing decrease in B cell number after acute clinical malaria infection [10], [39]. This may occur by either temporary reallocation of lymphocytes from peripheral circulation or by apoptosis [14], [33], [40]. Acute *Plasmodium* infection has also been shown to disrupt B cell lymphopoiesis in mouse models [41]. However, other studies have reported increased total B cell number in children with increase in intensity of malaria transmission [11] as well as severity of disease [42].

Interestingly, we noted increases in resting classical (CD19+IgD-CD27+CD21+) MBCs and concomitant decrease in activated (CD19+IgD-CD27+CD21-) MBCs. Chronic *Plasmodium falciparum* infection is a major cause of persistent polyclonal activation of B cells characterized by hypergammaglobulinemia [43] and increased production of autoantibodies [44]. The mechanism is thought to involve the cystein-rich interdomain region 1-α (CIDR1-α) of PfEMP1) [45].

We observed a significant increase in the marginal zone (MZ, (CD19+IgD+CD27+IgM+) B cells between 2008 and 2009. Human MZ B cells are a circulating pre-activated B cell subset thought to function primarily by controlling blood-borne pathogens [46]. Studies have demonstrated the presence of MZ B cells in patients lacking the ability to form germinal centers (GC) [24], leading to the suggestion they are not *bona fide* MBC. However, the findings that MZ B cells have diversified immunoglobulin genes albeit having a twofold lower mutation frequency compared to the isotype switched MBC [47] implies that they are a subset of MBCs. Furthermore, analysis of lymph nodes of hyper IgM patients has revealed abortive GC and GC-like structures [48]. This suggests that MZ B cells may be GC derived B cells that exit the GC reaction early before isotype switching occurs [13]. Nevertheless, blood and splenic MZ B cells in vaccinated children do not undergo antigen-induced clonal expansion [49]. Taken together, the evidence suggests that MZ B cells are a distinct MBC subset that develops and mutates early in life, independent of T-independent or T-dependent immune mechanisms [49].

Notably, there was a significant decrease in the class-switched atypical MBC. This subset may be related to a previously described population of CD27- non-classical MBC subset that normally resides in the spleen and tonsil and is very rare in peripheral circulation [50]. We did not assess CD10 and CD20 markers, so we do not know what fraction of the CD19+IgD-CD27-CD21- MBC observed in this study are the “exhausted” atypical MBC (CD19+CD10-CD27-CD21-) described in patients with high level HIV viremia [17] and in malaria-exposed individuals from other populations [12], [15], [16]. When the CD19+IgD-CD27-CD21 cells were examined for expression of the inhibitory FcRL4 receptor, an increase in the percentage that were FcRL4+ and also in the MFIs of FcRL4+ cells was seen between 2008 and 2009. A prior study found an increase in FcRL4+ cells in populations with heavy malaria transmission [12], so we would have expected a decrease in FcRL4 percentage and expression with the reduction of malaria transmission. The study participants had no increase in febrile illnesses over the time period of study, and none had clinical evidence of HIV or other chronic disease, so we have no obvious explanation for the increase in FcRL4+ cells over the time of study. Our future studies will examine whether changes in FcRL4 expression have any functional consequences on these B cell subsets.

The observed changes in B cell populations occurred concurrently with an absence of malaria episodes or detectable blood-stage infection among study participants. We surveyed the study participants and the health centers of the study area weekly for febrile illnesses, and did not find any outbreaks of new illnesses or diseases during this time period. We also assessed land use and were apprised of all Ministry of Health interventions and campaigns during the study period. Other than the spraying with indoor residual insecticide, no major new health initiatives were introduced during the study period, and no area wide distribution of medications or immunization outside of the standard national programs were done during this study period. Likewise, no changes in food supply or nutritional supplementation were recorded. Finally, cells from the two collection time points were assayed during the same time period, not separated by collection, and with the same flow cytometry parameters, so technical variations in sample testing cannot account for the differences seen. For all of these reasons, and with prior knowledge of the strong antigenic stimulation provided by malaria, we conclude that it is most likely that the changes seen in B cell populations were related to the absence of detectable or high-level *P. falciparum* infection in this population. However, future studies comparing across populations and over multiple time periods will be required to verify this hypothesis. A limitation of the study was that it may have failed to capture the asymptomatic infection that occurred between the 2–3 monthly fingerpick collections. However, even before interruption of malaria transmission, studies by our group have previously documented low prevalence of asymptomatic infections during a low transmission season at the site [51]. Nonetheless, it is possible that the presence of new antibodies in some individuals reflect detection of prior asymptomatic infection in a more sensitive manner than periodic testing by microscopy or PCR.

In summary, this study demonstrates that a one-year absence of detectable *P. falciparum* infection does not affect prevalence or levels of MSP-1_42_ specific antibodies or MBC in adults, but is associated with major changes in B cell homeostasis, notably a decrease in atypical and activate MBC, and an increase in resting classical MBC and in MZ B cells. These findings have implications for malaria vaccine studies as they suggest that acquired immune responses to malaria parasite antigens can be maintained in the absence of persistent infection but that the absence of repeated *P. falciparum* infection may lead to fundamental changes in overall B cell populations and memory B cell subsets. Additional studies are required to assess if the same changes occur in children, who are the main target population for a malaria vaccine. Future studies will further characterize memory B cells in populations of high and low transmission over time, and characterize antigen-specific MBC responses in these populations, to determine how changes in malaria transmission may affect responses to non-malarial antigens.

## Supporting Information

Figure S1 **A comparison of MBC subsets among participants with vs. without MSP1_42_-specific memory B cells in 2008.** There was no difference in the distribution of CD19+IgD-CD27-CD21+IgM-, CD19+IgD-CD27+CD21+IgM-, CD19+IgD-CD27+CD21-IgM- and CD19+IgD-CD27-CD21-IgM- B cells between the two groups.
(TIF)
