# Supplementary figures and images for: Changes in B Cell Populations and Merozoite Surface Protein-1-Specific Memory B Cell Responses after Prolonged Absence of Detectable P. falciparum Infection
Source: PLoS One. 2013 Jun 27;8(6):e67230. doi: 10.1371/journal.pone.0067230 (PMC3695086; doi:10.1371/journal.pone.0067230)

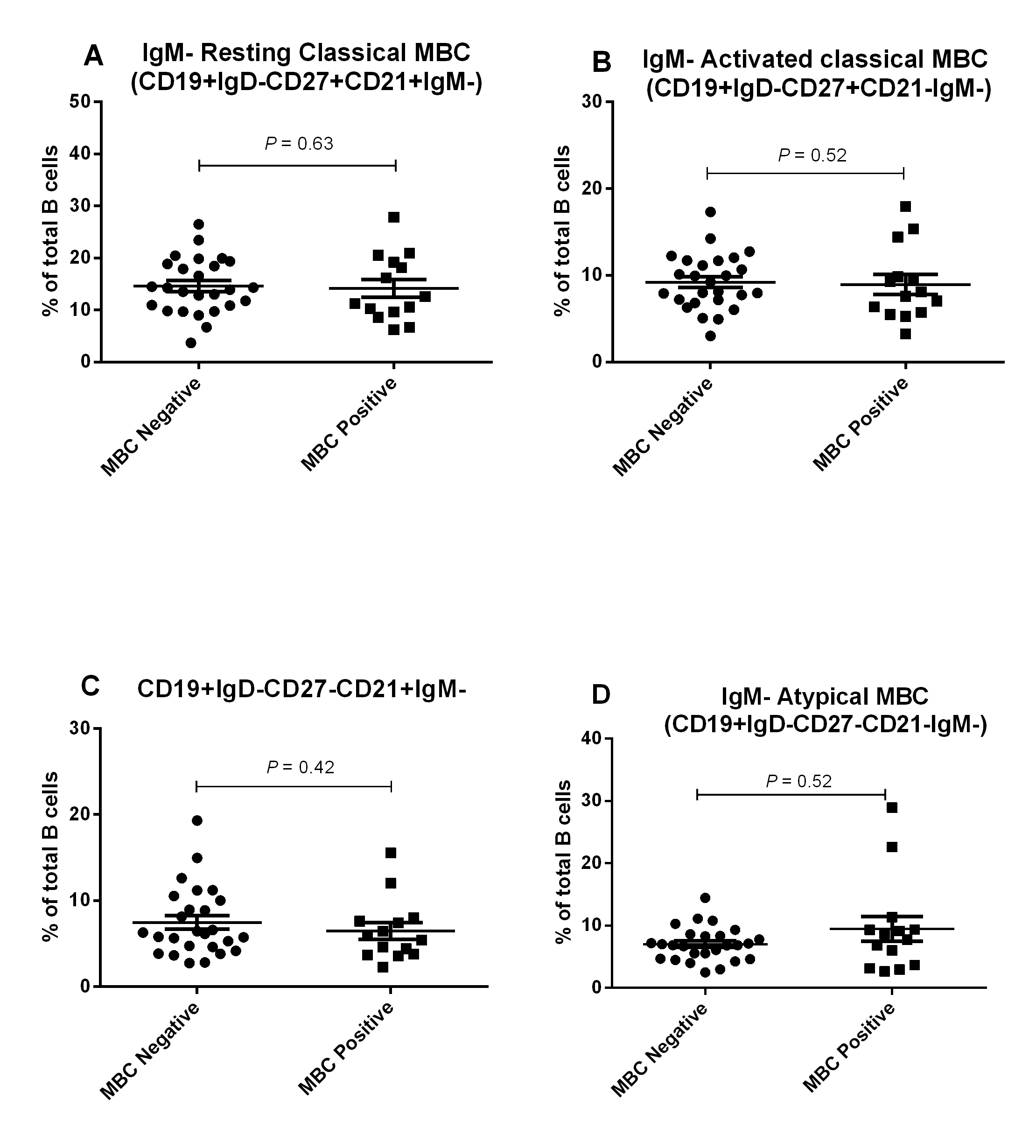

Supplement: Figure S1 — A comparison of MBC subsets among participants with vs. without MSP142-specific memory B cells in 2008. There was no difference in the distribution of CD19+IgD-CD27-CD21+IgM-, CD19+IgD-CD27+CD21+IgM-, CD19+IgD-CD27+CD21-IgM- and CD19+IgD-CD27-CD21-IgM- B cells between the two groups. (TIF) [file pone.0067230.s001.tif]
